# Radiographer gender and breast-screening uptake

**DOI:** 10.1038/sj.bjc.6604385

**Published:** 2008-05-27

**Authors:** P Fitzpatrick, A Winston, T Mooney

**Affiliations:** 1 BreastCheck, National Cancer Screening Service and UCD School of Public Health & Population Science, University College Dublin, Republic of Ireland; 2 BreastCheck, National Cancer Screening Service, Republic of Ireland

**Keywords:** breast screening, male radiographer, radiographer gender, uptake

## Abstract

BreastCheck, the Irish National Breast Screening Programme, screens women aged 50–64. Radiographer recruitment has been a challenge; doubling of numbers is required for full national expansion; to date females are employed. The aim was to document attitudes to male radiographers and effect on return for subsequent screening. In all 85.8% of a random sample of 2000 women recently screened by BreastCheck completed a postal questionnaire. The commonest reaction women felt they would have if there were a male radiographer was embarrassment; significantly greater among those attending a static unit (45.6%) than mobile (38.4%) and in younger women (46%) than older (38.7%). Nine per cent would not have proceeded if the radiographer was male and 9% would only have proceeded if female chaperone present. In all 17.5% (95% CI 15.7–19.4%) agreed that ‘If there were male radiographers I would not return for another screening appointment’; 18.3% were unsure. One-quarter agreed ‘if I heard there could be male radiographers it would change my opinion of BreastCheck for the worse’. The proportions agreeing with these statements did not vary significantly by screening unit type, age group, area of residence or insurance status. This is the largest published study to date of this important issue; the correct balance between equality and programme performance must be identified.

Despite its documented benefits many women view a mammogram with apprehension. A high level of breast consciousness, combined with modesty considerations and the possibility of an unfavourable diagnosis can leave many women feeling anxious and uncomfortable about having a breast examination. As a result, health care professionals have devoted attention and time to making mammography more accessible and more acceptable to greater numbers of women. Radiographer recruitment has been a challenge for BreastCheck, the National Breast Screening Programme in the Republic of Ireland; a doubling of numbers will now be required for national expansion, which commenced late 2007 (National Cancer Screening Service, 2007)

There has been very little research regarding women's attitudes to male radiographers for mammography, particularly in a screening setting as opposed to symptomatic. The aim of this study was to document attitudes to male radiographers among women screened by BreastCheck, to compare attitudes by screening unit, area of residence and key socio-demographic indicators, and to specifically examine how employment of male radiographers could affect participation in the screening programme. The overall eligible women uptake rate in BreastCheck is 78.1% (73.1% for first invitees, 18% for previous non-attenders and 90% for subsequent invitees) (National Cancer Screening Service, 2007).

## MATERIALS AND METHODS

A postal questionnaire was sent to a random sample of 2000 women recently screened by BreastCheck (in 2006), each of whom had an outcome of normal mammogram. Women who have been recalled for further investigation were not included. An information sheet was enclosed. Informed consent was implied by return of the completed questionnaire. A freepost envelope was included for return of questionnaire. Questionnaires were anonymous; a second questionnaire was sent to all subjects to increase response rate.

Respondents were asked to indicate the initial reaction(s) they would likely experience if there was a male radiographer present for screening, and to choose one of four possible statements to best reflect how they would have proceeded if there was a male radiographer. Using a Likert scale respondents were asked to indicate agreement with a series of statements regarding screening and attitudes to radiographer gender.

### Sample size calculation

By sending out 2000 questionnaires (with estimated final response rate of 75%) we sought to achieve the required sample size of 1500 (which was exceeded), which would allow detection of an overall proportion of negative attitude to male radiographers to within ±2% (with 80% power at the 5% significance level) and for any comparisons detection of differences in attitudes between groups of approximately 6% (e.g. between mobile and static screening units).

### Statistical analysis

Statistical analysis was performed using the SAS statistical package. Proportions were compared between age groups, between those with and without private medical insurance, those screened in a static and mobile unit and those from urban and rural residence. The *χ*^2^ test was used for comparison of proportions. Confidence intervals (95%) are presented for overall proportions.

### Ethical approval

The study was approved by the Research Ethics Committee of the Faculty of Public Health Medicine, Royal College of Physicians of Ireland.

## RESULTS

A total of 1716 women responded, giving a response rate of 85.8%. In all 33.2% were aged 50–54 years, 33.5% aged 55–59 years, 30.5% aged 60–64 years and 2.9% were over 64 years. Overall 23.3% of respondents held a medical card and 63.8% had private insurance. A total of 43.9% lived in a rural/countryside location, 32.3% in a town and 23.8% in a city. In all 62.4% had been screened in a mobile unit, the remainder in a static unit. For 273 their recent BreastCheck mammogram was their first mammogram, whereas 1408 had had a previous mammogram. The responders reflected well those women who attended for screening, where uptake is higher among upper social classes and similar numbers are screened from each age group. The predominance of women screened in a mobile unit reflects the ratio of mobile to static unit screening carried out by the programme.

### Initial reaction

Overall the most common reaction women felt they would have if there was a male radiographer was embarrassment (Table 1). The proportions indicating embarrassment were statistically significantly greater among women screened in a static unit than in a mobile unit (45.6 *vs* 38.4%; *P*=0.003) and in younger women (50–54 years) compared to older (46 *vs* 38.7%; *P*=0.004).

One-third of all respondents indicated they would have no reaction; proportions were significantly greater among rural than urban women (36.6 *vs* 31.6%; *P*=0.03) A significantly greater proportion of women screened in a mobile unit than in static indicated they would have no reaction (36.1 *vs* 29.2%; *P*=0.004).

A further third of all respondents indicated surprise as the initial reaction. Small proportions of women indicated their initial reaction would be annoyance or anger.

### Proceed with mammogram?

Respondents were asked to tick one of a number of statements to best reflect how they would have proceeded if there was a male radiographer (Table 1). The largest number of respondents stated they would have proceeded to have their mammogram performed but would have preferred a female radiographer. Almost 9% would not have proceeded if the radiographer was male and a further 9% would only have proceeded if there were a female chaperone present.

Overall there was a significant difference in responses from women with and without private medical insurance regarding how they would proceed, with greater proportions of women with private insurance than without stating they would have refused to have mammogram performed if radiographer was male (9.6 *vs* 7.4%) or that they would have preferred a female chaperone (48.3 *vs* 39.4%). There was no significant difference between those attending static and mobile units or between those living in urban and rural settings with regard to how they would proceed.

Approximately one-third of women in each age group indicated they would have proceeded to have their mammogram performed if there was a male radiographer. The proportions indicating they would have refused to have the mammogram performed if the radiographer was male or that they would have had mammogram performed only if there was a female chaperone did not differ significantly between age groups.

### Attitudes to screening and radiographer gender

Overall, 17.5% (95% CI 15.7–19.4%) agreed or strongly agreed with the statement ‘If there were male radiographers I would not return to BreastCheck for another screening appointment’; a further 18.3% were unsure. In all 16.2% of those aged 50–54 years, 15.7% of those aged 55–59 years and 19.8% of those aged 60–64 years agreed/strongly agreed that they would not return. Overall almost one-quarter agreed or strongly agreed with the statement ‘if I heard there could be male radiographers it would change my opinion of BreastCheck for the worse’. Almost half agreed or strongly agreed that it would be very embarrassing having a male radiographer for breast screening. However, overall the majority of women (78%; 95% CI 76–80%) agreed/strongly agreed with the statement ‘having my breast screening performed is more important to me than any concern about the gender of staff dealing with me’. The proportions agreeing with these statements did not vary significantly by type of screening unit, age group, area of residence or insurance status (Table 2).

Overall over 40% agreed/strongly agreed that that they would be equally comfortable with a male as with a female radiographer for breast screening, one-third disagreed/strongly disagreed and a further 20.9% were unsure. A significantly smaller proportion of women screened at a static unit than at a mobile unit (37.8 *vs* 46.0%; *P*=0.001), a smaller proportion of younger than older women (37.9 *vs* 45.4%; *P*=0.004) and a smaller proportion of women with private insurance than without (50.3 *vs* 38.1%; *P*<0.0001) agreed/strongly agreed with this statement.

Overall 62.8% agreed/strongly agreed with the statement ‘Gender would not matter – the only relevant issue is how good a radiographer is at his/her job’. A significantly smaller proportion of women screened at a static unit than at a mobile unit (56.8 *vs* 66.7%; *P*<0.0001), of younger (58.4%) than older women (58.4 *vs* 65.3%; *P*=0.006), of women living in an urban area than rural area (66.1 *vs* 60.5%; *P*=0.02) and of women with private insurance than without (58.6 *vs* 70.1%; *P*<0.0001) agreed or strongly agreed with this statement.

### Attitudes to radiographer and surgeon gender for follow-up investigations

Overall over 90% agreed/strongly agreed that the gender of a surgeon doing follow up investigations to confirm or out rule a breast cancer would not matter; a statistically significant smaller proportion (75.8%; *P*<0.0001) agreed/strongly agreed that the gender of a radiographer would not matter. The proportions agreeing that the gender of the radiographer would not matter differed significantly between rural women and urban women (79.5 *vs* 73.0%, *P*=0.002) and between women with and without private insurance (73.9 *vs* 78.8%; *P*=0.002).

### Gender preference for radiographer and surgeon in future

Respondents were asked ‘what would be your preference regarding the gender of the radiographer who performs your next mammogram?’; the majority (60%, 95% CI 57.6–62.3%) opted for a female radiographer, 3.1% for a male and 36.9% had no preference. A statistically significant greater number of women in the 50–54 year age group indicated a preference for a female radiographer for the next mammogram compared to women aged over 54 (64.4 *vs* 58.1%; *P*=0.01) and in women with private medical insurance than those without (64.0 *vs* 53.9%; *P*<0.0001).

When asked, ‘if you had to have breast surgery at some time in the future, what would be your preference regarding the gender of the surgeon who would perform your surgery?’ the majority (81.5%, 95% CI 79.6–83.3%) had no preference, 10.8% opted for a female and 7.7% for a male. These proportions did not differ significantly when compared between age groups, screening units, residence or insurance status.

## DISCUSSION

Among the advantages of this study are the large sample size, the random sampling technique employed and the excellent response rate. The sample size is the largest of any published study on this subject. Although with a postal questionnaire a very high response rate is not usually expected, with the target population a sample of recently screened women with a normal outcome, it was felt that a 75% response rate could be attained; this was surpassed, with a response rate of 85.8%.

Adequate uptake of breast screening is imperative to achieve the mortality reduction sought (Perry *et al*, 2006). The findings of this study suggest that attitudes to male radiographers for screening would reduce uptake rates. Looking at the situation internationally in screening programmes similar to the Irish model, the NHS Breast Screening Programme do not recruit male radiographers for breast screening (unpublished data); in the Swedish breast screening programme a small number of male radiographers are employed in some rural centres, but radiographers are predominantly female (unpublished data), while in the Dutch breast screening programme all radiographers are female (unpublished data).

Most previous published work comes from the USA, where the structure of screening programmes differs from that employed in Europe and much of the research has been conducted in the symptomatic setting. Much of the published work was conducted in the early 1990s. In a study conducted at a Denver clinic 75% of 180 women said they would not object to a qualified male mammographer (Greer, 1993). A further study in New Jersey showed that 50% of women objected to male student involvement in mammography (Tilke, 1993). A study in Norway found that of 178 women who failed to obtain mammograms, 3% indicated the possibility of having a male examiner as their reason for non-attendance (Gram and Slenker, 1992). In this study 17.5% of women indicated they would not return for screening if there were male radiographers.

In this study 60% expressed a preference for female mammographers. This compares to 82.3% in a study in the USA conducted in medical offices, hospitals and obstetrics and gynaecology clinics (Serbus, 1994). In the US study one in six women had no preference compared to 30% in this study. Almost one-third of women in the US study said they would reschedule the examination with a female radiographer if initially offered a male radiographer compared with 17.7% of women in this study who would refuse to proceed if there were a male radiographer or would insist on a female chaperone.

These findings are similar to those found where attitudes to male nurses for intimate examination has been studied, where younger women in particular prefer a female nurse (Chur-Hansen, 2002). Degree of intimacy in interaction has been found to be a persistent predictor of same-gender preferences for nurses (Chur-Hansen, 2002). Female patient preference for female midwives has been found to be stronger than female preference for any other health professional type (Kerssens *et al*, 1997).

Changing feminine culture would lend credence to the notion that the impact of having male radiographers would lessen over time. However, this is not reflected fully in this study, as a slightly greater proportion of women in the youngest age group stated they would not return compared to women in the middle age group.

The response to radiographer gender was less marked when further investigations to out rule or confirm a cancer were in hand. This tallies to some extent with the greater proportions positively disposed to male mammographers found in symptomatic studies. In Ireland historically most surgeons were male, and although this is changing, women would still frequently expect a male surgeon.

The findings of this study are of importance for all those screening for breast cancer. The correct balance between equality and programme performance must be identified.

## Figures and Tables

**Table 1:** Initial reaction and indication of how one would proceed

**Reaction**	***N* (%)**	**95%CI**
Embarrassment	703 (41.0%)	38.6–43.3%
No reaction	577 (33.6%)	31.4–35.9%
Surprise	519 (30.3%)	28.1–32.5%
Annoyance	86 (5.0%)	4.1–6.2%
Anger	36 (2.1%)	1.5–2.9%
Pleased	20 (1.2%)	0.7–1.8%
		
*Statement re proceeding*		
I would have had my mammogram by the male radiographer but would have preferred a female radiographer or to have a female chaperone with me	752 (44.9%)	42.5–47.3%
I would have had my mammogram if there was a male radiographer	627 (37.4%)	35.1–39.8%
I would have had my mammogram performed but only if there was a female chaperone with the male radiographer	149 (8.9%)	7.5–10.3%
I would have refused to have the mammogram performed if the radiographer was male	148 (8.8%)	7.5–10.3%

**Table 2:** Agreement with statements regarding attitudes to screening and radiographer gender

**Statement**	**Strongly agree**	**Agree**	**Unsure**	**Disagree**	**Strongly disagree**
I would be equally comfortable with a male radiographer as with a female radiographer for breast screening	224 (13.4%)	489 (29.3%)	348 (20.9%)	383 (23.0%)	223 (13.4%)
Gender would not matter – the only relevant issue is how good a radiographer is at his/her job	496 (29.9%)	544 (32.9%)	199 (12.0%)	294 (17.8%)	123 (7.4%)
Having my breast screening performed is more important to me than any concern about the gender of staff dealing with me	743 (44.6%)	558 (33.5%)	144 (8.6%)	155 (9.3%)	6 (4.0%)
It would be very embarrassing having a male radiographer for breast screening	280 (17.0%)	464 (28.1%)	222 (13.5%)	449 (27.2%)	234 (14.2%)
If there were male radiographers I would not return to BreastCheck for another screening appointment	121 (7.5%)	163 (10.0%)	298 (18.3%)	588 (36.1%)	457 (28.1%)
If I heard there could be male radiographers it would change my opinion of BreastCheck for the worse	160 (9.7%)	241 (14.7%)	231 (14.1%)	594 (36.2%)	415 (25.3%)

## References

[bib1] Chur-Hansen A (2002) Preferences for female and male nurses: the role of age, gender and previous experience – year 2000 compared with 1984. J Adv Nurs 37: 192–198 11851787 10.1046/j.1365-2648.2002.02079.x

[bib2] Gram I, Slenker S (1992) Cancer anxiety and attitudes toward mammography among screening attenders, non-attenders and women never invited. Am J Public Health 82: 249–251 1739156 10.2105/ajph.82.2.249 PMC1694295

[bib3] Greer K (1993) Attitudes toward the male mammographer. Advance for Rad Science Professionals 5: 5–11

[bib4] Kerssens JJ, Bensing JM, Andela MG (1997) Patient preference for genders of health professionals. Soc Sci Med 44: 1531–1540 9160442 10.1016/s0277-9536(96)00272-9

[bib5] National Cancer Screening Service (2007) BreastCheck Annual Report 2006/07. National Cancer Screening Service: Dublin

[bib6] Perry N, Broeders M, De Wolf C, Tornberg S, Holland R, von Karsa L (2006) European Guidelines for Quality Assurance in Breast Screening and Diagnosis, 4th edn. Office for Official Publications of the European Communities: Luxembourg 10.1093/annonc/mdm481 18024988

[bib7] Serbus C (1994) Survey of women's opinions of male mammographers. Radiol Technol 65: 220 8127956

[bib8] Tilke B (1993) Educators bringing mammography education up-to-date. Advance for Rad Science Professionals 6: 13–46

